# Specific human antibody responses to *Aedes aegypti* and *Aedes polynesiensis* saliva: A new epidemiological tool to assess human exposure to disease vectors in the Pacific

**DOI:** 10.1371/journal.pntd.0006660

**Published:** 2018-07-24

**Authors:** Françoise Mathieu-Daudé, Aurore Claverie, Catherine Plichart, Denis Boulanger, Fingani A. Mphande, Hervé C. Bossin

**Affiliations:** 1 UMR MIVEGEC, IRD, CNRS, UM, Institut de Recherche pour le Développement, Nouméa, Nouvelle-Calédonie; 2 UMR MIVEGEC IRD, CNRS, UM, Institut de Recherche pour le Développement, Montpellier, France; 3 Pôle de recherche et de veille sur les maladies infectieuses émergentes, Institut Louis Malardé, Papeete, Tahiti, Polynésie française; 4 Laboratoire d’entomologie médicale, Institut Louis Malardé, Paea, Tahiti, Polynésie française; North Carolina State University, UNITED STATES

## Abstract

**Background:**

*Aedes* mosquitoes severely affect the health and wellbeing of human populations by transmitting infectious diseases. In French Polynesia, *Aedes aegypti* is the main vector of dengue, chikungunya and Zika, and *Aedes polynesiensis* the primary vector of Bancroftian filariasis and a secondary vector of arboviruses. Tools for assessing the risk of disease transmission or for measuring the efficacy of vector control programmes are scarce. A promising approach to quantify the human-vector contact relies on the detection and the quantification of antibodies directed against mosquito salivary proteins.

**Methodology/Principal findings:**

An ELISA test was developed to detect and quantify the presence of immunoglobulin G (IgG) directed against proteins from salivary gland extracts (SGE) of *Ae*. *aegypti* and *Ae*. *polynesiensis* in human populations exposed to either species, through a cross-sectional study. In Tahiti and Moorea islands where *Ae*. *aegypti* and *Ae*. *polynesiensis* are present, the test revealed that 98% and 68% of individuals have developed IgG directed against *Ae*. *aegypti* and *Ae*. *polynesiensis* SGE, respectively. By comparison, ELISA tests conducted on a cohort of people from metropolitan France, not exposed to these *Aedes* mosquitoes, indicated that 97% of individuals had no IgG directed against SGE of either mosquito species. The analysis of additional cohorts representing different entomological *Aedes* contexts showed no ELISA IgG cross-reactivity between *Ae*. *aegypti* and *Ae*. *polynesiensis* SGE.

**Conclusions/Significance:**

The IgG response to salivary gland extracts seems to be a valid and specific biomarker of human exposure to the bites of *Ae*. *aegypti* and *Ae*. *polynesiensis*. This new immuno-epidemiological tool will enhance our understanding of people exposure to mosquito bites, facilitate the identification of areas where disease transmission risk is high and permit to evaluate the efficacy of novel vector control strategies in Pacific islands and other tropical settings.

## Author summary

In Pacific islands like in most tropical regions, *Aedes* mosquitoes affect the health of human populations by transmitting diseases like dengue, chikungunya, Zika and filariasis. The biting nuisance of *Aedes* mosquitoes also impacts local tourism, affecting the sustainability of island economies. Mosquito saliva is injected during the biting process, and the response triggered by the human immune system to proteins contained in mosquito saliva was shown to be a relevant biomarker of exposure to mosquito bites. Using this approach, we have developed an immuno-epidemiological tool to investigate the exposure of people to the bites of *Aedes aegypti* and *Aedes polynesiensis*, two significant mosquito vectors of infectious diseases in French Polynesia and other island countries and territories in the Pacific. This novel tool proved specific and reliable. It will improve the assessment of disease transmission risk and be useful for measuring the efficacy of both conventional and innovative vector control strategies.

## Introduction

In the Pacific island countries and territories (PICTs), *Aedes* mosquitoes severely affect the health and wellbeing of local communities by transmitting infectious pathogens, mainly arboviruses (dengue, chikungunya, Zika, Ross River) and parasites (the filaria *Wuchereria bancrofti*). *Aedes* mosquitoes are also a major nuisance for local tourism, affecting the sustainability of island economies. Located in the South Pacific, French Polynesia includes 74 populated islands with Tahiti, the largest island encompassing nearly 70% of the whole population [1]. The four serotypes of DENV have prompted successive epidemics recorded since the 1940s [2–8], and outbreaks due to Zika (ZIKV) and chikungunya (CHIKV) viruses have also been reported recently [4, 9, 10]. The epidemiology of DENV in French Polynesia, as in several other PICTs, is characterized by the persistence of a single serotype with an endemic pattern of transmission for 4–5 years until the virus causes a new outbreak or is replaced by another serotype [5, 6, 11, 12]. Thus, the last dengue outbreak, initiated in 2013 continues in 2017. In 2013–2014, Zika virus caused a severe outbreak in French Polynesia with 49% disease prevalence rates and asymptomatic:symptomatic case ratios (1:1) in the general population [13]. While Zika virus infection was previously described as a relatively mild disease consisting of fever, rash, arthralgia, headache, and conjunctivitis [14, 15], this ZIKV outbreak of an unprecedented magnitude was characterized by severe neurologic complications, such as Guillain-Barré syndrome in adults [16, 17] and microcephaly in fetuses and newborns [18].

On the parasitological side, infection with *Wuchereria bancrofti*, an helminth responsible for a disfiguring lymphatic filariasis (LF) mainly in rural habitats, is still of public health importance in some PICTS even though progress towards achieving LF elimination has been made through the Pacific Programme for the Elimination of Lymphatic Filariasis (PacELF) [19, 20]. In French Polynesia, despite several rounds of mass drug administration (MDA) from 2000 to 2007, the overall prevalence in the population was 11.3% in 2008 [21]. Following enhanced efforts since then, the overall prevalence has dropped, but French Polynesia continues implementation of MDA coverage in areas displaying residual transmission [20].

Present in most PICTs *Aedes aegypti* (Diptera, Culicidae) is the major vector of dengue virus in the South Pacific [22]. Native to the South Pacific, the Polynesian tiger mosquito *Aedes polynesiensis*, is the main vector of lymphatic filariasis and a secondary vector of dengue. It has also been involved in Ross River virus transmission in Tahiti [10, 23, 24]. Besides French Polynesia, it is found in abundance in Fiji, Wallis and Futuna, Tuvalu, Kiribati, Tokelau, Samoa, American Samoa, Cook Islands, and Pitcairn. Vector competence investigations showed that both *Aedes* species could transmit chikungunya and Zika, under laboratory conditions [25, 26]. The mostly anthropophilic *Ae*. *aegypti* is commonly seen in urban habitats such as coastal cities and villages whereas *Ae*. *polynesiensis* is more frequently observed in the valleys and selvatic biotopes. Both are daytime blood feeders, with peak biting times in the early morning and late afternoon. Climatic conditions in the Society Islands, which include Tahiti and Moorea, are characterized by a warm and wet season from October through May (Austral summer) and a relatively dryer and cooler season from November through April (Austral winter). Seasonality impacts mosquito population density with *Ae*. *aegypti* and *Ae*. *polynesiensis* populations reaching highest abundance during the wet season. However, seasonal density dynamics is higher for *Ae*. *polynesiensis* than *Ae*. *aegypti*, the latter being more dependent on domestic larval habitats [27].

Strategies aimed at preventing or reducing vector-borne diseases must take into account both pathogen and vector. There is no routine vaccine nor therapeutic treatment against either arbovirus found in French Polynesia and annual mass-drug administration campaigns targeting lymphatic filariasis, are costly and time-consuming. Therefore, the prevention of human contacts with mosquitoes remains central to control disease transmission. Human exposure to mosquito bites and the efficacy of vector control interventions are usually extrapolated from entomological methods based on larval and/or adult sampling. However, these labor-intensive methods present substantial limitations, owing to the cost of their implementation, and generate poorly suited indicators for the prediction of disease transmission and the risk of outbreaks [28]. The development of complementary methods and indicators to evaluate the actual human exposure to *Aedes* bites is a necessary step to improve vector control strategies and to assess the risk of disease transmission.

As reviewed by Doucoure and Drame [29], human antibody (Ab) responses to arthropod salivary proteins were shown to be relevant biomarkers of human exposure to mosquito bites. In the *Aedes* genus, anti-saliva antibodies proved to be a valuable immuno-epidemiological tool for evaluating exposure to *Aedes albopictus* bites in Reunion island [30] and to *Ae*. *aegypti* bites in Bolivia [31], Thaïland [32] and Colombia [33].

Novel vector control strategies based on the release of incompatible *Aedes* males carrying the endosymbiotic bacterium *Wolbachia* to suppress mosquito populations are under field evaluation in French Polynesia [34]. The efficacy of this *Wolbachia*-based vector control approach could be better evaluated by monitoring changes in exposure of human populations to *Aedes* mosquito bites in treated areas.

The main objective of our study conducted in French Polynesia was to evaluate the potential of human IgG responses against *Ae*. *aegypti* and *Ae*. *polynesiensis* saliva as biomarkers of exposure to mosquito bites in Tahiti and Moorea islands where residents are naturally exposed to both *Aedes* species. An additional group of military personnel who had not been exposed to the bites of these two mosquito species prior to their arrival in Tahiti was tested twice, upon arrival and after a year of residence, in order to follow the Ab responses. Complementary cohorts from various *Aedes* exposure contexts served as controls to help differentiating cross-reactions.

## Materials and methods

### Study design and populations

A cross-sectional study was performed on a population of French Polynesian residents and on five other human cohorts selected for their diverse exposures to *Aedes* mosquito species. No travel outside their respective area of main residence within six months prior to blood sampling was a pre-requisite to the enrollment of study participants. An additional group of French Polynesian military personnel sampled twice, a year apart, was included in the study.

The origin, sample size and main characteristics of the study populations are summarized in Table 1. For the five cohorts originating from outside French Polynesia, blood or serum was received as dried spots on filter paper (Serobuvard, LDA22, Ploufragan, France) to ensure sample stability upon shipment [9]. The French Polynesian cohort from Tahiti and Moorea islands was collected by Institut Louis Malardé (Tahiti, French Polynesia) on adults aged 18 years and older, during the Austral summer. The sera from the military personnel were collected in 1994 by Institut Louis Malardé two weeks upon their arrival in Tahiti and then a year later, most of them (11/13) during the Austral summer, and kept frozen. Additional blood or serum samples were collected in 2012 by Institut Pasteur in New Caledonia (Southwest Pacific Ocean) at the beginning of the cool season, and by the French Blood Bank in two overseas French islands: Martinique (French West Indies) at the end of the hot and rainy season, and Reunion (Indian Ocean) at the end of the Austral summer (hot and wet season). Regarding the Reunion cohort, half of the samples (17/33) originated from a previous study [30]. These sera were collected in May-June 2009 during the seasonal peak of *Ae*. *albopictus* exposure, from adults residing in the south of Reunion island [30]. The cohort from Bolivia (South America) originated from an urban area in the city of Santa Cruz de la Sierra. Sera were collected by the Centro Nacional de Enfermedades Tropicales during a multidisciplinary dengue study, during the hot and wet season when *Ae*. *aegypti* was abundant [31]. This Bolivian cohort was the only one to include children, 15/30 (50%) under 18 years old with 7 of them under 14 (23%). Samples from metropolitan France were collected during the winter season (February 2012) by the French Blood Bank in Paris and Strasbourg (eastern France) from adults aged 18 years and older.

**Table 1:** Characteristics of the populations under study.

Sampling location	Population	n	Age*	Sex ratio**	Sampling year	Blood sample	Pattern of exposure	
							*Ae*. *polynesiensis*	*Ae*. *aegypti*
Metropolitan France	Residents	66	>18	NA	2012	Serum, dried spot	-	-
Tahiti and Moorea, French Polynesia	Residents	47	>18	NA	2012	Serum	+	+
Martinique (French West Indies)	Residents	46	>18	NA	2012	Serum, dried spot	-	+
New Caledonia (South Pacific)	Residents	19	42 (25–57)	47%	2012	Blood, dried spot	-	+
Bolivia	Residents	30	24 (6–64)	67%	2007	Serum, dried spot	-	+
Reunion (Indian Ocean)	Residents	33	26 (18–30)	52%	2009 2012	Serum, dried spot	-	- ^c^
Tahiti, French Polynesia	Military personnel	13	31 (22–39)	69%	1993^a^	Serum, frozen	-	-
Tahiti, French Polynesia	Military personnel	13	31 (22–39)	69%	1994^b^	Serum, frozen	+	+

* median age (range) when available; >18 means adult cohort

** expressed as female %

^a^ upon arrival

^b^ after a year of residence

^c^ cryptic species in Reunion, not present in the area where human blood samples were collected

NA, data not available

+ indicates the mosquito presence, and – indicates its absence.

### Cohorts exposure to *Aedes* vectors

The cohorts patterns of exposure to *Ae*. *aegypti* and *Ae*. *polynesiensis* bites are shown in Table 1. In Tahiti and Moorea islands (French Polynesia), cohorts are diversely exposed to both *Aedes* species [25, 35]. *Ae*. *polynesiensis* is not present in Martinique, New Caledonia nor Bolivia where *Ae*. *aegypti* is the major *Aedes* species [22, 31, 36]. In Reunion island, *Ae*. *albopictus* is abundant, while *Ae*. *aegypti* is cryptic [30, 37] and *Ae*. *polynesiensis* is absent. In metropolitan France, *Ae*. *aegypti* and *Ae*. *polynesiensis* are not present and *Aedes albopictus* was not present in the sampled areas (Paris and North East of France) at the time of sampling in 2012 [38]. Other *Aedes* species, some of them anthropophilic, are present in these regions of metropolitan France, and people living there might have been exposed to these *Aedes* species, not at the time of sampling in winter, but during the previous summer.

### Collection of *Ae*. *aegypti* and *Ae*. *polynesiensis* salivary gland extracts (SGE)

*Ae*. *aegypti* (Bora-Bora strain) and *Ae*. *polynesiensis* (Moorea strain) were reared in the insectarium of the Medical Entomology Laboratory, Institut Louis Malardé (Tahiti, French Polynesia) using standard procedures (26°C ± 2°C, ambient photoperiod, 80% relative humidity). Three day-old female mosquitoes were blood fed on mice, and two days later sedated at 4°C for 10 minutes for salivary gland dissection. Fifty pairs of salivary glands were pooled in 100 μl of phosphate buffered saline (PBS) with 1/100 dilution of protease inhibitor mix (GE Healthcare, United-Kingdom) and frozen at -20°C before protein extraction. Salivary glands were grinded and homogenised using a mixer mill (Retsch, Haan, Germany) and one bead (4 mm diameter) per tube at 20 Hz for 2 minutes. Homogenates were then centrifuged at 12 000 g for 5 minutes at 4°C and soluble proteins were quantified using Bradford Reagent (Sigma-Aldrich, Saint-Louis, MO, USA). Protein quantification was estimated at 1.88 μg and 1.20 μg per salivary gland pair for *Ae*. *aegypti* and *Ae*. *polynesiensis*, respectively. Aliquots of SGE in PBS were stored at -20°C.

### Evaluation of human antibody responses to *Aedes* SGE

An enzyme-linked immunosorbent assay (ELISA) was used to detect and evaluate the level of human antibody circulating in blood and directed against *Ae*. *aegypti* or *Ae*. *polynesiensis* SGE. Dried blood spots were eluted overnight at 4°C in blocking buffer (PBS-0.5% Tween 20–10% goat serum (Sigma-Aldrich)). Spots corresponding to 20 μl of blood were eluted in 780 μl of buffer, giving a concentration of eluted proteins equivalent to 1:40 dilution of the original blood sample, and approximately 1:80 dilution with respect to serum, assuming a hematocrit of 50% [39]. Maxisorp microplates (Nunc, Roskilde, Denmark) were coated for 2.5 hours at 37°C with 100 ng of SGE from the two *Aedes* species separately. After two washes with 300 μl of wash buffer (PBS-0.1% Tween 20) plates were blocked with 300 μl of blocking buffer for 45 minutes at 37°C. After two washes, plates were incubated overnight at 4°C with 100 μl per well of individual serum (1:80 dilution in blocking buffer) or eluted blood sample (1:40 dilution, equivalent to 1:80 dilution of the serum in the sample). A 1:3000 dilution of biotinylated mouse anti-human IgG (BD Biosciences, San Jose, CA, USA) in blocking buffer was added to each well (100 μl per well) and incubated for 1.5 hour at 37°C. Plates were washed three times and 100 μl of peroxidase-conjugated streptavidin (GE Healthcare Amersham, Little Chalfont, UK) diluted at 1:3000 in blocking buffer was added to each well. After one hour incubation at 37°C followed by four washes, revelation was performed by adding 100 μl of ABTS solution (Roche, Basel, Switzerland). Absorbance was read one hour later in a spectrophotometer at 405 nm and optical density (OD) values were recorded. Each serum or blood sample was treated in duplicate (OD_1_ and OD_2_) and in a blank well containing no SGE (OD_b_) to measure non-specific binding, as previously described [40]. Individual result was expressed as the ΔOD calculated using the equation: ΔOD = [(OD_1_ + OD_2_) / 2]—OD_b_. An individual was considered as an “immune responder” if the ΔOD was above the cut-off value calculated as (mean of ΔODs from unexposed individuals) + (3 x Standard Deviation value) [30].

### Statistical analysis

Data were analyzed using GraphPad Prism version 7.0 (GraphPad Software Inc., La Jolla CA, USA). After confirmation of a non-normal distribution, a non-parametric test, Mann-Whitney (two independent groups), Wilcoxon matched paired (two paired groups) or Kruskal-Wallis (more than 2 independent groups) tests were used. Dunn's multiple comparison test was used for bivariate comparison of multiple medians. All differences were considered significant at p-value (*p*) <0.05.

### Ethics statement

All studies followed ethical principles as stipulated in the Edinburgh revision of the Helsinki Declaration. Studies were approved by the Ethics Committee of French Polynesia (opinion No. 52, March 2012), the Bolivian committee of Bioethics (September 2006), the Institut de Recherche pour le Développement (IRD) “*Comité consultatif de Déontologie et d’Ethique*” (July 2006), the French Drug Agency (AFFSAPS, Ministry of Health, January 2009) and the French South-West and Overseas Regions Ethics Committee (February 2009). Written informed consent was obtained from every volunteer and parents or legal guardians provided consent on behalf of child participants. A number was assigned to each participant to ensure anonymity.

## Results

### IgG responses against *Ae*. *aegypti* and *Ae*. *polynesiensis* SGEs in French Polynesia

**Fig 1:**
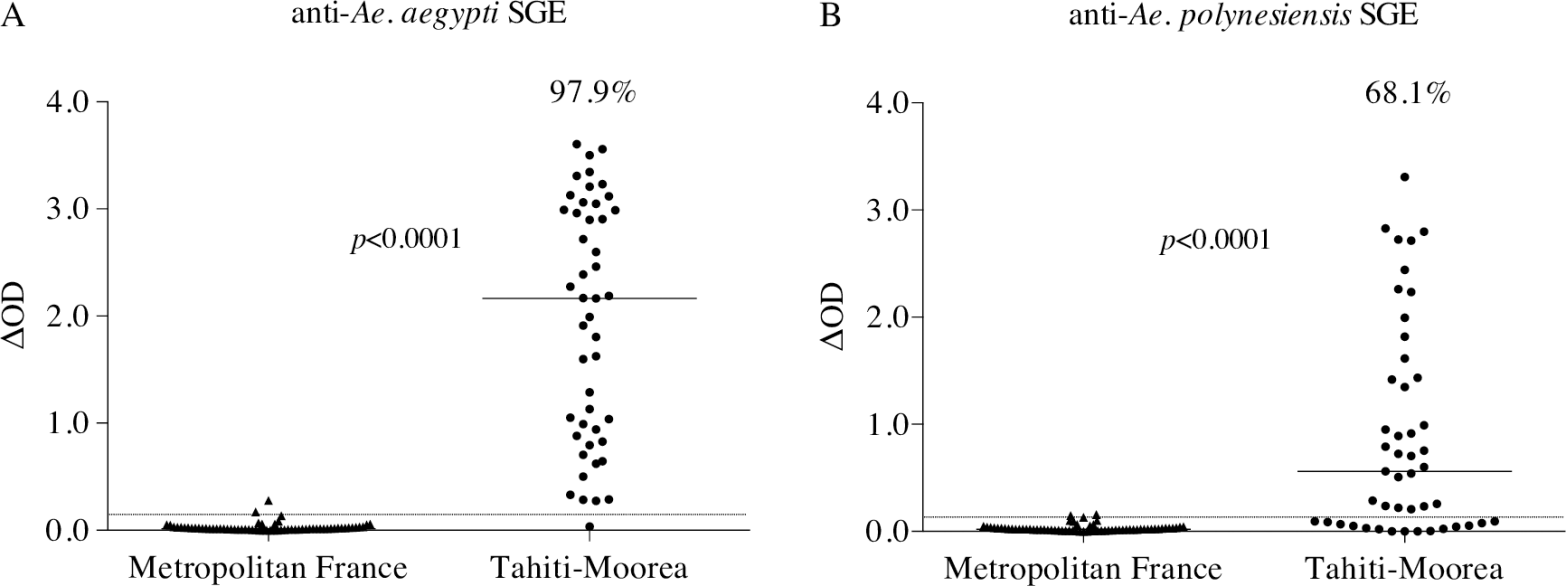
Antibody responses to *Ae*. *aegypti* and *Ae*. *polynesiensis* SGE in French Polynesian residents. The figure presents the individual IgG responses (ΔOD) against *Ae*. *aegypti* (A) and *Ae*. *polynesiensis* (B) SGE in metropolitan France (n = 66) and in Tahiti and Moorea islands (French Polynesia, n = 47) residents. Each triangle or dot represents an individual sample and the horizontal bar indicates the median value. The dotted lines correspond to the positivity thresholds calculated from the cohort of metropolitan French residents, not exposed to these *Aedes* species (0.16 for *Ae*. *aegypti* and 0.13 for *Ae*. *polynesiensis*). The percentage of responders (positive samples) in the Tahiti-Moorea cohort is shown above the plot. The non-parametric Mann-Whitney test was used to compare groups.

IgG responses directed against *Ae*. *aegypti* and *Ae*. *polynesiensis* SGEs were evaluated using ELISA on sera from Tahiti and Moorea islands where both *Ae*. *aegypti* and *Ae*. *polynesiensis* are abundant in their respective habitats. French metropolitan serum samples were tested as negative controls and their ΔOD values allowed the determination of the cut-off value for sample positivity as described. This positivity threshold was 0.16 and 0.13 for *Ae*. *aegypti* and *Ae*. *polynesiensis*, respectively (Fig 1). Thus, two out of 66 tested metropolitan samples were positive against either *Ae*. *aegypti* or *Ae*. *polynesiensis* (values just above threshold), giving a negativity rate of 97.0%. Among the Tahiti-Moorea Polynesian cohort only one out of 47 samples was considered negative to *Ae*. *aegypti* SGE (OD < 0.16), giving an overall 97.9% positivity rate (*p*<0.0001) (Fig 1A). However, within positive subjects, ODs varied greatly in magnitudes, ranging from 0.28 to 3.61. IgG positive responses against *Ae*. *polynesiensis* SGE were observed in 68.1% of the tested samples (OD > 0.13) (*p*<0.0001), with individual ODs ranging from 0.21 to 3.31 (Fig 1B). Globally, the Tahiti-Moorea cohort showed higher reactivity to *Ae*. *aegypti* SGE compared to *Ae*. *polynesiensis* SGE both in terms of positivity rates (97.9% versus 68.1%) and of median values (2.16 versus 0.56).

### Antibody kinetics in a naive cohort exposed to *Ae*. *aegypti* and *Ae*. *polynesiensis* in Tahiti island (French Polynesia)

**Fig 2:**
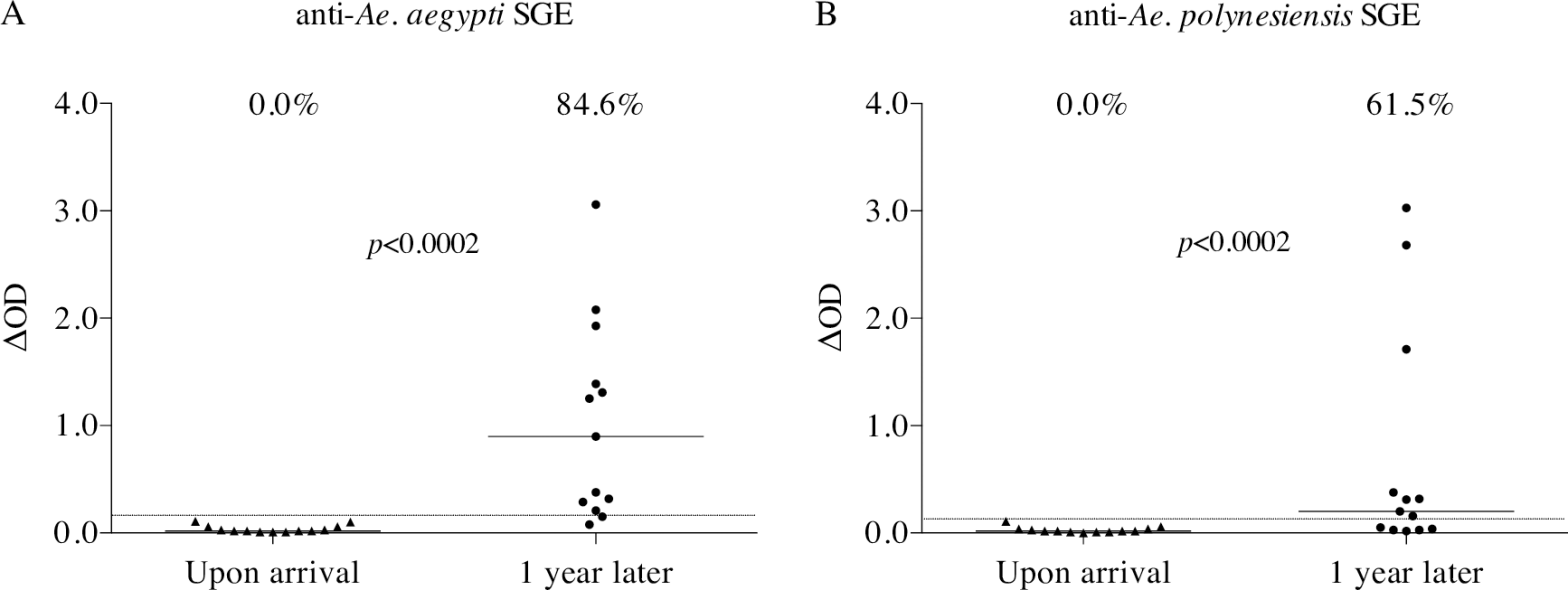
Evolution of antibody responses to *Ae*. *aegypti* and *Ae*. *polynesiensis* SGE in military personnel. The figure presents individual IgG responses (ΔOD) to *Ae*. *aegypti* (A) and *Ae*. *polynesiensis* (B) SGE of French military personnel (n = 13) assigned in Tahiti island (French Polynesia). Blood from the same individual was sampled two weeks after arrival and one year later (paired data). Each triangle or dot represents an individual serum and the horizontal bar indicates the median value. The dotted lines correspond to the positivity thresholds calculated from the cohort of metropolitan French residents (0.16 and 0.13 for *Ae*. *aegypti* and *Ae*. *polynesiensis*, respectively). Percentages of responders are shown above each plot. The non-parametric Wilcoxon test was used to compare the paired groups.

Upon arrival, none of the 13 military personnel had specific anti-SGE IgG antibodies (Fig 2). After one year of continuous stay in Tahiti island, 11 of them (84.6%, *p*<0.0002) became positive against *Ae*. *aegypti* and 8 (61.5%, *p*<0.0002) against *Ae*. *polynesiensis* SGEs. Of the negative responders, only one did not respond to SGE of both *Aedes* species. Similar to the cohort of Tahiti-Moorea residents, individual IgG levels were heterogeneous within the positive military personnel, ranging from 0.21 to 3.06 for *Ae*. *aegypti* SGE (Fig 2A) and from 0.16 to 3.03 for *Ae*. *polynesiensis* SGE (Fig 2B). The median IgG values significantly increased between both sampling times for both *Aedes* species.

### IgG responses to *Ae*. *aegypti* and *Ae*. *polynesiensis* SGEs in Martinique, New Caledonia Bolivia and Reunion cohorts

**Fig 3:**
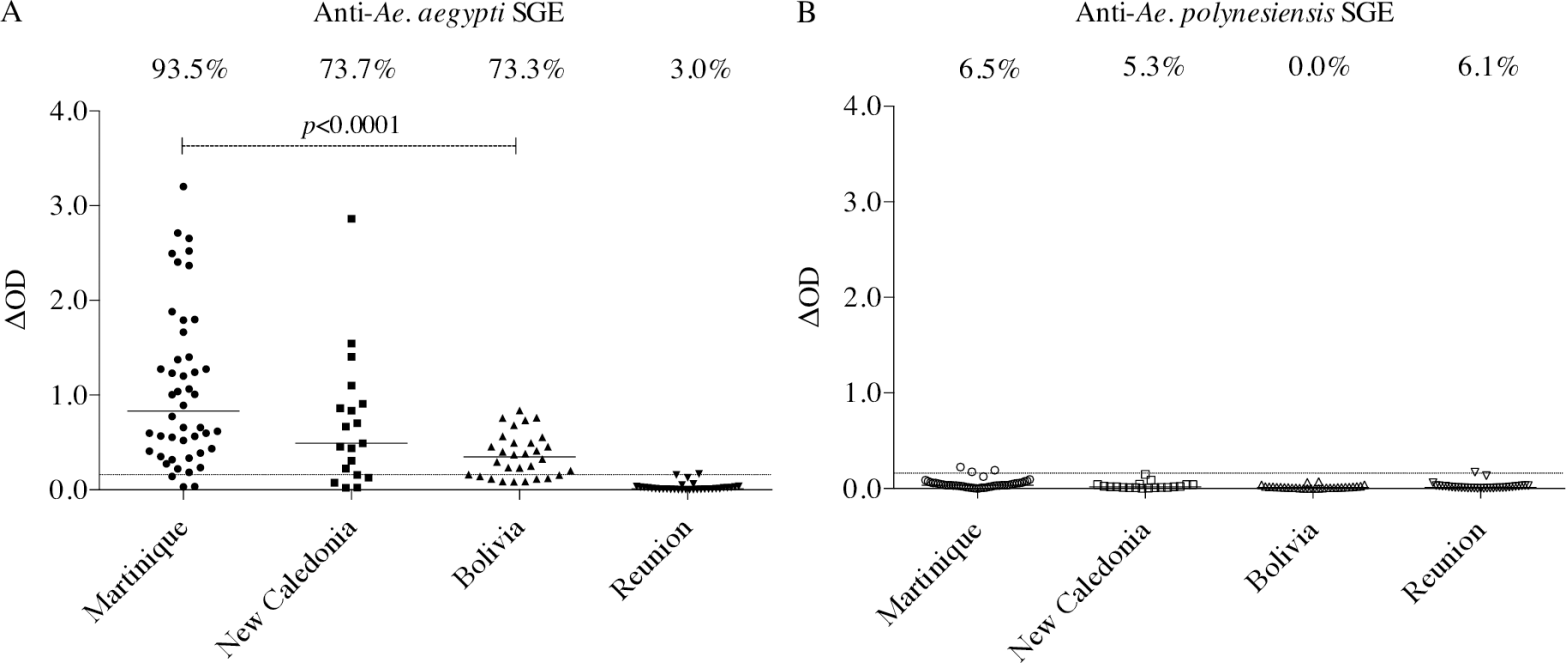
Antibody responses to *Ae*. *aegypti* and *Ae*. *polynesiensis* SGE from cohorts from different contexts of exposure. The figure presents the individual IgG responses (ΔOD) against *Ae*. *aegypti* (A) and *Ae*. *polynesiensis* (B) SGE of residents from Martinique (n = 46), New-Caledonia (n = 19), Bolivia (n = 30) and Reunion (n = 33). The horizontal bars indicate the median value in each group and the dotted lines correspond to the positivity thresholds calculated from the cohort of metropolitan French residents. The percentage of responders in each cohort is shown above the plot. Residents from Martinique, New Caledonia and Bolivia, are typically exposed to *Ae*. *aegypti* bites. In Reunion island, *Ae*. *albopictus* is the main *Aedes* species while *Ae*. *aegypti* is cryptic. *Ae*. *polynesiensis* is not present in these islands or countries.

Blood and serum samples from the three cohorts of Martinique, New Caledonia and Bolivia showed reactivity to *Ae*. *aegypti* SGE. Median IgG levels and rates of positivity to *Ae*. *aegypti* SGE were as follows: Martinique (0.83; 93.5%), New-Caledonia (0.49; 73.7%), Bolivia (0.35; 73,3%) (Fig 3A). When comparing both the median values and the percentages of positivity to *Ae*. *aegypti* SGE, the cohort from Martinique showed the highest reactivity, followed by New Caledonia and Bolivia cohorts. Difference between these cohorts was significant between Martinique and Bolivia (*p*<0.0001). But for these three cohorts, IgG response positivity to *Ae*. *polynesiensis* SGE was (i) rare with an overall rate of positivity of 4.2% (4/95), (ii) of very low magnitude (maximum of 0.23; cut-off = 0.13) (Fig 3B). In Reunion island, IgG responses developed by residents against either *Ae*. *aegypti* or *Ae*. *polynesiensis* SGE were very low (Fig 3A and 3B). Only 1 out of 33 residents was considered positive, at a background level of 0.17 (cut-off value = 0.16) for *Ae*. *aegypti*; and 2 out of 33 at a background level of 0.14 and 0.17 (cut-off value = 0.13) for *Ae*. *polynesiensis*, giving positivity rates of 3.0% and 6.1% for the two species, respectively. Regarding the IgG responses to *Ae*. *aegypti* SGE (Fig 3A), the differences between Reunion and each of the three other cohorts were significant (p<0.0001). Part of the Reunion cohort (17/33 individuals) originated from a previous work [30]. These individuals were all detected as positive responders who had developed anti-*Ae*. *albopictus* SGE IgG. The median value of the IgG level of this subsample of the original cohort was 2.32 (individual values ranging from 1.67 to 3.14, positivity threshold was 0.27). This subsample thus represents our positive controls for the exposure and response to *Ae*. *albopictus* SGE among the Reunion cohort.

## Discussion

Vector-borne diseases in French Polynesia are mainly characterized by (i) the circulation of major arboviruses (dengue, chikungunya and Zika) [4], (ii) the presence of a crippling helminthiasis (Bancroft’s filariasis) [21], and (iii) the presence of two *Aedes* vector species (*Ae*. *aegypti* and *Ae*. *polynesiensis*) [35, 41]. Control of the filarial parasite is under progress but the increasing frequency and severity of arbovirus outbreaks over the last decade warrants for heightened vector control measures. To identify indicators of actual human exposure to *Aedes* bites, we conducted in the Tahiti and Moorea islands a serological screening of IgG responses to *Aedes* mosquito saliva. Similar indicators have been evaluated in different entomological and epidemiological contexts [29]. The present study reports the detection of IgG Ab directed against proteins from SGE of *Ae*. *aegypti* and *Ae*. *polynesiensis* in human populations exposed to these vectors. To the best of our knowledge, this is the first study examining the status of anti-mosquito saliva Ab in a Pacific island cohort. Moreover, before this study the immunogenicity of *Ae*. *polynesiensis* salivary proteins was completely unknown.

In Tahiti and Moorea islands, our data showed a very high (97.9%) or high (68.1%) sensitization of the resident island community towards *Ae*. *aegypti* and *Ae*. *polynesiensis* saliva, respectively. Strikingly, the individual specific Ab levels were highly heterogeneous, in accordance with what is repeatedly observed when using the same type of antigen (*Aedes* SGE) in metropolitan France [42], Reunion island [30], Bolivia [31] and Colombia [33, 43]. These results suggest that both immunogenicity of salivary proteins and/or levels of exposure vary between individuals for a given *Aedes* species. Indeed, intensity of Ab response elicited by salivary antigens may vary between individuals according to their immune system [44]. Moreover, individuals are not homogeneously bitten in a population, since the spatial distribution of *Aedes* mosquito vectors is commonly heterogeneous in disease endemic areas [45], and people are not exposed in the same way to mosquito bites because of their differences in behavior (*i*.*e*., mosquito source reduction, use of protective clothing or mosquito repellents) and their variable attractiveness to mosquitoes, through emissions of carbon dioxide and volatile organic compounds produced by the human body including skin microbiota [46, 47]. All these differences can explain the high heterogeneity observed in Ab response levels in a population.

Due to its “naive” nature, since none of the military personnel had specific anti-SGE Ab upon arrival in Tahiti, the cohort of military personnel gave some useful clues regarding the pattern of seroconversion: 84.6% and 61.5% became positive towards the saliva of, respectively, *Ae*. *aegypti* and *Ae*. *polynesiensis*, strongly suggesting that bites from these *Aedes* species abundant in Tahiti elicited the development of acquired anti-saliva Ab responses. The percentages of seroconversion after a year were much higher than that observed after a 5-month journey in tropical Africa where only 15% of a military population showed significantly increased IgG responses against *Ae*. *aegypti* saliva antigens [48]. It is interesting that several soldiers remained negative against *Ae*. *polynesiensis*, while most turned positive to *Ae*. *aegypti* after a year in Tahiti. This pattern is consistent with the differential distribution of these two *Aedes* species on the islands of Tahiti and Moorea, with *Ae*. *aegypti* being abundant around human dwellings in urban areas and villages and *Ae*. *polynesiensis* most present at the periphery in valleys, and forested areas [35]. Thus people are not exposed in the same way to these two vector species according to their places of residence and daily activities. For these individuals who did not exhibit IgG reponse to *Ae*. *polynesiensis* saliva antigens, their professional or personal habits would likely not have exposed them to *Ae*. *polynesiensis* bites sufficient to elicit a detectable Ab response.

However, IgG Ab responses to *Ae*. *aegypti* and *Ae*. *polynesiensis* SGE represent responses to two different sets of antigens, more precisely two different complex mixtures of salivary proteins, harbouring different immunogenic properties. Therefore we can not quantitatively compare the intensities of the Ab responses to *Ae*. *aegypti* versus *Ae*. *polynesiensis* and deduce a differential exposure to either species.

For a given mosquito species, intensities of IgG Ab responses can be compared between cohorts, or between different sampling times of a cohort study, and previous surveys have repeatedly demonstrated that IgG response medians provided a reliable estimate of the average level of cohorts exposure to mosquito bites [30, 42, 49]. Their follow-up through space and over time would thus provide valuable insights into the development of immunity of the different cohorts to the bites of each *Aedes* species.

In the present work, most cohorts were composed of adults aged 18 years and older, to avoid possible bias linked to immunity development with aging. Immunity development in a population with aging was previously investigated by comparing IgG levels against *Ae*. *aegypti* salivary proteins between age groups in an urban cohort from Bolivia [31]. In this publication, the authors reported significantly higher IgG levels in children (<14 years of age) and a progressive decrease in IgG response with aging. However, the authors could not conclude whether this difference in IgG responses to mosquito saliva reflected children being exposed to a greater number of *Aedes* bites, a stronger immune reaction of children to *Aedes* bites, or a progressive desensitization of adults to salivary proteins with aging. In the present work, the Bolivian cohort was the only one to include children. We tested possible differences between three age groups (<14, 14 to 18, >18; Kruskal-Wallis test) or between two age groups (<18, >18; Mann-Whitney test) and we did not observe significant differences. It would be interesting in the future to study how immunity develops among age groups in cohorts exposed to either *Ae*. *aegypti* or *Ae*. *polynesiensis*.

Blood or serum spots collected onto filter paper are an established and convenient source of antibodies for serological diagnosis and epidemiological surveys [39, 50]. However different types of biological samples were used in the present study raising potential bias concerns. For the New Caledonia cohort, elution of whole blood spots was adjusted taking into account the hematocrit percentage [39] and the antibody recovery was assumed to be efficient in all sample types as previously described [51, 52]. Stability of IgG antibodies from dried whole blood or serum on filter paper has also been reported [51]. In the present work antibodies recovery from dried serum spots proved to be efficient since the Martinique cohort displayed high IgG responses. Other potential concerns were alleviated by comparing paired samples of fresh vs dried serum deposited on filter paper (<4 weeks) from a subsample of the Tahiti-Moorea cohort. No significant differences in the Ab response were detected. Further assessment of the recovery and quality of Ab should be performed nonetheless through the quantification of IgG response to *Aedes* salivary proteins from paired samples of fresh blood or serum, vs dried blood or serum spots on filter paper. Same type of biological samples should be used in the future for quantitative comparisons of IgG responses between cohorts.

To investigate the possibility of cross-reactive epitopes between salivary proteins, we screened both *Aedes* species SGE with sera collected in four different locations where *Ae*. *polynesiensis* was absent. In three of them (Martinique, New-Caledonia, Bolivia), *Ae*. *aegypti* was present but not in the fourth (Reunion island) where *Ae*. *albopictus* is the main anthropophilic *Aedes* species while *Ae*. *aegypti* is cryptic [37]. The Reunion cohort was exposed to *Ae*. *albopictus* bites at the time of sampling and half of the samples have been previously tested and found positive responders to *Ae*. *albopictus* SGE [30]. Analysis of sera from *Ae*. *aegypti*-exposed areas showed no clear cross-reactions with *Ae*. *polynesiensis* SGE, and that of sera from Reunion showed no cross-reactions between *Ae*. *albopictus* and either *Ae*. *aegypti* or *Ae*. *polynesiensis* SGE. Taken together, these data suggest that the IgG Ab response observed is species-specific and that the immunogenic proteins expressed in the sialome of the *Aedes* genus differ sufficiently between the different species under study, at least regarding the epitopes. This is in agreement with previous observations made in Reunion island where only weak cross-reactivity was detected between *Ae*. *albopictus* and *Ae*. *aegypti* SGE [30].

A salivary peptide, the Nterm-34kDa peptide present on the immunogenic 34kDa-salivary protein of *Ae*. *aegypti*, has been designed as a specific biomarker of *Ae*. *aegypti* bites and proved to be a useful tool to evaluate human exposure to *Ae*. *aegypti* [53]. However, a subsequent study indicated that IgG Ab response to this salivary peptide was equally relevant for evaluating the efficacy of vector control interventions against another *Aedes* species, *Ae*. *albopictus*, in Reunion island [54]. These results pointed out that IgG response to the *Aedes* Nterm-34kDa salivary peptide could be used as a general biomarker of *Aedes* bites, at the genus level but not at the species level. The low cross-reactivity between the Ab responses to SGE of the different *Aedes* species observed in [30] and in the present work would however indicate that the abundant and/or immunogenic proteins are sufficiently different between these species to present different epitopes, and thus should represent a promising asset for the development of species specific peptides. Therefore, efforts are being pursued to develop salivary peptides truly specific of each *Aedes* vector species (*Ae*. *aegypti*, *Ae*. *albopictus*, *Ae*. *polynesiensis*), targeting specific epitopes on salivary proteins.

Given their heterogeneity, IgG responses may not be used to assess the risk of disease at the individual level. These studies however, are useful at a group or population level. Indeed results on Ab responses from cohorts can inform us on spatial differences or temporal variations in exposure to mosquito bites (*e*.*g*., natural seasonal variations or reduction of exposure resulting from successful vector control interventions). Although such biomarkers do not directly assess infective bites they provide a meaningful assessment of exposure to mosquito vectors, and indirectly therefore to a risk of infection. The evaluation of human IgG response to mosquito salivary proteins can thus help identify areas with heightened risks of arbovirus transmission. In the present work, no epidemiological or medical data were available at country level that could be associated with the levels of Ab response to *Aedes* salivary proteins observed in the study subjects. However, IgG biomarkers of exposure to mosquito salivary proteins have been confirmed as useful proxies of dengue and malaria infections in several studies [43, 55, 56].

Results of the present work unequivocally demonstrate that bites from both *Aedes* species elicit a marked humoral reactivity in local residents of French Polynesia. This reactivity is strongly supported by the results of the military personnel follow-up where most individuals developed specific antibodies against *Ae*. *aegypti* and *Ae*. *polynesiensis* during their stay in Tahiti. This study represents the first attempt to detect an antibody response to salivary proteins of *Ae*. *polynesiensis* in exposed populations. The specificity of the responses to *Ae*. *aegypti* and *Ae*. *polynesiensis* SGE offers great potential as a specific biomarker of exposure to these species for epidemiological studies in French Polynesia as in other settings where both species are present.

Therefore, their detection and follow-up in the blood of human population will provide useful indicators to identify areas where people are subject to potentially infectious *Aedes* bites. It will help to target vector control strategies against filariasis and major arboviroses such as dengue, chikungunya or Zika. Coupled with classical entomological indices [57], this ELISA assay should also prove useful for monitoring the efficacy of vector control procedures including novel *Wolbachia*-based mosquito suppression strategies under evaluation in French Polynesia.

## Supporting information

S1 Checklist STROBE checklist. (DOC)
