# Supplementary material for: Specific human antibody responses to Aedes aegypti and Aedes polynesiensis saliva: A new epidemiological tool to assess human exposure to disease vectors in the Pacific
Source: PLoS Negl Trop Dis. 2018 Jul 24;12(7):e0006660. doi: 10.1371/journal.pntd.0006660 (PMC6075770; doi:10.1371/journal.pntd.0006660)
Supplement: S1 Checklist — (DOC) [file pntd.0006660.s001.doc]

STROBE Statement—Checklist of items that should be included in reports of ***cross-sectional studies***

|  | Item No | Recommendation |
| --- | --- | --- |
| **Title and abstract** | 1 | (*a*) Indicate the study’s design with a commonly used term in the title or the abstract [in **Abstract, Methodology/Principal Findings]** |
|  |  | (*b*) Provide in the abstract an informative and balanced summary of what was done and what was found [in **Abstract, Methodology/Principal Findings** and **Conclusions/Significance]** |
| Introduction | | |
| Background/rationale | 2 | Explain the scientific background and rationale for the investigation being reported  [in **Introduction, background lines 68-112; rationale lines 113-134]** |
| Objectives | 3 | State specific objectives, including any prespecified hypotheses [in **Introduction, lines 135-142]** |
| Methods | | |
| Study design | 4 | Present key elements of study design early in the paper [in **Material and methods, Study design and populations, lines 146-150]** |
| Setting | 5 | Describe the setting, locations, and relevant dates, including periods of recruitment, exposure, follow-up, and data collection [in **Material and methods, paragraph Study design and populations lines 151-172,** and **paragraph Cohorts exposure to Aedes vector, lines 184-194;** and in **Table 1]** |
| Participants | 6 | (*a*) Give the eligibility criteria, and the sources and methods of selection of participants [in **Material and methods, Study design and populations, lines 146-149]** |
| Variables | 7 | Clearly define all outcomes, exposures, predictors, potential confounders, and effect modifiers. [exposures/predictors in **Material and methods, paragraph Cohorts exposure to Aedes vector, lines 185-194;** and in **Table 1;** outcomes (ODs) in **paragraph Evaluation of human antibody responses to Aedes SGE, lines 212-229]**  Give diagnostic criteria, if applicable [**Not applicable]** |
| Data sources/ measurement | 8* | For each variable of interest, give sources of data and details of methods of assessment (measurement). Describe comparability of assessment methods if there is more than one group. [For exposures, data given in **Material and methods, paragraph Cohorts exposure to Aedes vector, lines 185-194 including the references;** and in **Table 1;** outcomes (ODs) with methods of measurement in **paragraph Evaluation of human antibody responses to Aedes SGE, lines 229-236]** |
| Bias | 9 | Describe any efforts to address potential sources of bias [inclusion of different populations described in **Introduction, lines 138-142** and in **Material and methods, paragraph Study design and populations, lines 146-150]** |
| Study size | 10 | Explain how the study size was arrived at [in **Material and methods, paragraph Study design and populations, lines 151-172;** and in **Table 1]** |
| Quantitative variables | 11 | Explain how quantitative variables were handled in the analyses. If applicable, describe which groupings were chosen and why [in **Material and methods, Evaluation of human antibody responses to Aedes SGE, lines 233-236]** |
| Statistical methods | 12 | (*a*) Describe all statistical methods, including those used to control for confoundingwhy [in **Material and methods, Statistical analysis, lines 238-244]** |
|  |  | (*b*) Describe any methods used to examine subgroups and interactions [**Not applicable]** |
|  |  | (*c*) Explain how missing data were addressed [**Not applicable]** |
|  |  | (*d*) If applicable, describe analytical methods taking account of sampling strategy [**Not applicable]** |
|  |  | (*e*) Describe any sensitivity analyses [**Not applicable]** |
| Results | | |
| Participants | 13* | (a) Report numbers of individuals at each stage of study—eg numbers potentially eligible, examined for eligibility, confirmed eligible, included in the study, completing follow-up, and analysed [Initial numbers given in **Material and methods, Table 1;** and analyzed numbers (identical) are given in the **Results** section**,** and **in the Figure legends, lines 258-338]** |
|  |  | (b) Give reasons for non-participation at each stage [**Not applicable]** |
|  |  | (c) Consider use of a flow diagram [**Not applicable]** |
| Descriptive data | 14* | (a) Give characteristics of study participants (eg demographic, clinical, social) and information on exposures and potential confounders [previously presented in **Material and methods, paragraphs Study design and populations,** and **Cohorts exposure to Aedes vector, lines 151-194;** and in **Table 1.** Additional information is provided in the **Results** section**, lines 321-327]** |
|  |  | (b) Indicate number of participants with missing data for each variable of interest [**Not applicable]** |
| Outcome data | 15* | Report numbers of outcome events or summary measures [in **Results,** and **Figure legends, lines 258-338,** and on **Figures 1 to 3]** |
| Main results | 16 | (*a*) Give unadjusted estimates [in **Results,** and **Figure legends, lines 258-338]**  and, if applicable, confounder-adjusted estimates and their precision (eg, 95% confidence interval) [**Not applicable]**. Make clear which confounders were adjusted for and why they were included [**Not applicable]** |
|  |  | (*b*) Report category boundaries when continuous variables were categorized [**Not applicable]** |
|  |  | (*c*) If relevant, consider translating estimates of relative risk into absolute risk for a meaningful time period [**Not applicable]** |
| Other analyses | 17 | Report other analyses done—eg analyses of subgroups and interactions, and sensitivity analyses [**Not applicable]** |
| Discussion | | |
| Key results | 18 | Summarise key results with reference to study objectives [in **Discussion, lines 341-354]** |
| Limitations | 19 | Discuss limitations of the study, taking into account sources of potential bias or imprecision. Discuss both direction and magnitude of any potential bias [in **Discussion, lines 388-392, 399-412, 413-427]** |
| Interpretation | 20 | Give a cautious overall interpretation of results considering objectives, limitations, multiplicity of analyses, results from similar studies, and other relevant evidence [in **Discussion, lines 355-370, 371-387, 429-456 and 458-470]** |
| Generalisability | 21 | Discuss the generalisability (external validity) of the study results [End of **Discussion, lines 472-487]** |
| Other information | | |
| Funding | 22 | Give the source of funding and the role of the funders for the present study and, if applicable, for the original study on which the present article is based [Funding information are not given **in the manuscrit but provided during the submission process, according to the journal’s submission guidelines]** |

*Give information separately for exposed and unexposed groups.

**Note:** An Explanation and Elaboration article discusses each checklist item and gives methodological background and published examples of transparent reporting. The STROBE checklist is best used in conjunction with this article (freely available on the Web sites of PLoS Medicine at http://www.plosmedicine.org/, Annals of Internal Medicine at http://www.annals.org/, and Epidemiology at http://www.epidem.com/). Information on the STROBE Initiative is available at www.strobe-statement.org.
